# Increased incidence of traffic accidents in *Toxoplasma*-infected military drivers and protective effect RhD molecule revealed by a large-scale prospective cohort study

**DOI:** 10.1186/1471-2334-9-72

**Published:** 2009-05-26

**Authors:** Jaroslav Flegr, Jiří Klose, Martina Novotná, Miroslava Berenreitterová, Jan Havlíček

**Affiliations:** 1 Department of philosophy, Faculty of Science, Charles University, Vinièná 7, Prague 128 44, Czech Republic; 2 Central Medical Psychology Department, Central Military Hospital, U vojenské nemocnice 1200, Prague 169 02, Czech Republic; 3 Department of Anthropology, Faculty of Humanities, Charles University, Husnikova 2075, Prague 158 00, Czech Republic

## Abstract

**Background:**

Latent toxoplasmosis, protozoan parasitosis with prevalence rates from 20 to 60% in most populations, is known to impair reaction times in infected subjects, which results, for example, in a higher risk of traffic accidents in subjects with this life-long infection. Two recent studies have reported that RhD-positive subjects, especially RhD heterozygotes, are protected against latent toxoplasmosis-induced impairment of reaction times. In the present study we searched for increased incidence of traffic accidents and for protective effect of RhD positivity in 3890 military drivers.

**Methods:**

Male draftees who attended the Central Military Hospital in Prague for regular entrance psychological examinations between 2000 and 2003 were tested for *Toxoplasma *infection and RhD phenotype at the beginning of their 1 to1.5-year compulsory military service. Subsequently, the data on *Toxoplasma *infection and RhD phenotype were matched with those on traffic accidents from military police records and the effects of RhD phenotype and *Toxoplasma *infection on probability of traffic accident was estimated with logistic regression.

**Results:**

We confirmed, using for the first time a prospective cohort study design, increased risk of traffic accidents in *Toxoplasma*-infected subjects and demonstrated a strong protective effect of RhD positivity against the risk of traffic accidents posed by latent toxoplasmosis. Our results show that RhD-negative subjects with high titers of anti-*Toxoplasma *antibodies had a probability of a traffic accident of about 16.7%, i.e. a more than six times higher rate than *Toxoplasma*-free or RhD-positive subjects.

**Conclusion:**

Our results showed that a common infection by *Toxoplasma gondii *could have strong impact on the probability of traffic accident in RhD negative subjects. The observed effects could provide not only a clue to the long-standing evolutionary enigma of the origin of RhD polymorphism in humans (the effect of balancing selection), but might also be the missing piece in the puzzle of the physiological function of the RhD molecule.

## Background

A protozoan parasite *Toxoplasma gondii *infects 20–60% of the population in most countries, depending on climate, hygienic standards and cooking habits [1]. Postnatally acquired toxoplasmosis in immunocompetent subjects causes mild disease, acute toxoplasmosis, which turns spontaneously into lifelong latent toxoplasmosis. Latent toxoplasmosis is characterized by the presence of the dormant cyst stage of the parasite mainly in the neural and muscular tissues and immunity against new *Toxoplasma *infections [2,3]. Latent toxoplasmosis in humans is considered as clinically asymptomatic [4,5]. However, infected people have impaired reaction times [6] and about 2.6 times higher risk of traffic accidents [7,8], possibly as a result of manipulation activity of *Toxoplasma *aimed to increase the chance of transmission from the intermediate to the definitive host, i.e. from any bird or mammal species to any feline species, by predation.

Recently, two studies on two populations of blood donors, one of conscripts [9] and the other of university students [10], have shown that RhD-positive subjects, and RhD heterozygotes in particular, are protected against latent toxoplasmosis-induced impairment of reaction times. The RhD protein which is the *RHD *gene product and a major component in the Rh blood group system carries the strongest blood group immunogen, the D antigen. The structure homology data suggest that the RhD protein acts as an ion pump of uncertain specificity and unknown physiological role [11,12]. The D antigen is absent in a significant minority of the human population (RhD-negatives) due to *RHD *deletion or alternation. For a long time, the origin of this RhD polymorphism was an evolutionary enigma. Before the advent of modern medicine, the carriers of the rarer allele (e.g. RhD-negative mutants in the population of RhD-positives or RhD-positive mutants in the population of RhD-negatives) were at a disadvantage as some of their children (RhD-positive children born to preimmunised RhD-negative mothers) were at a higher risk of foetal or newborn death or health impairment from haemolytic disease. The higher tolerance of RhD-positive heterozygotes against *Toxoplasma*-induced impairment of reaction time could counterbalance the disadvantage of the rarer allele and could be responsible both for the initial spread of the RhD allele among the RhD-negative population and for a stable RhD polymorphism in most human populations. Differences in the prevalence of *Toxoplasma *infection between geographical regions could also explain the striking variation in the frequency of RhD-negative alleles between populations.

A real impact of the latent toxoplasmosis-associated impairment of reaction times on human life (and therefore also a possible toxoplasmosis-associated selection pressure) was estimated by comparison of the prevalence of toxoplasmosis among victims of traffic accidents and control samples of people living in the same areas in two independent studies, in Prague (Czech Republic), and Izmir and Manisa (Turkey) [7,8]. However, they were both designed as retrospective case-control studies. Therefore, it was impossible to tell whether *Toxoplasma*-infected subjects had a higher risk of traffic accidents or whether *Toxoplasma*-free subjects had a higher chance to be included into epidemiological surveys used to estimate the prevalence of toxoplasmosis in the general population (the value used for controls in case-control studies), for example because of their higher superego strength and lower suspiciousness [13]. Moreover, the RhD status of victims and controls was not monitored and included in statistical analyses of the obtained data. The aims of the present study were 1) to test whether *Toxoplasma*-infected subjects have a higher risk of traffic accidents or a lower probability of being enrolled in epidemiological surveys and 2) to search for possible protective effect of RhD phenotype against traffic accidents in *Toxoplasma*-infected subjects. For these purposes we tested for latent toxoplasmosis and RhD phenotype a large population of conscript drivers in the beginning of their 12–18-month military service and estimated the risk of traffic accidents in different subpopulations of conscripts, using military police records.

## Methods

### Subjects

A study population comprised of 3890 male draftees (mean age 20.1 years, s.d. 1.55) who attended the Central Military Hospital in Prague for regular entrance psychological examinations between 2000 and 2003 and consented to participate in the research project. The draftees were tested for *Toxoplasma *infection and RhD phenotype at the beginning of their 1 to1.5-year compulsory military service. The overall seroprevalence of *Toxoplasma *infection was 29.7% and 22.8% of subjects were RhD-negative. In the Czech general male population of comparable age, the seroprevalence of *Toxoplasma *infection would be approximately 28.7% and the rate of RhD-negative subjects would be approximately 18% [14]. It must be stressed out, however, that the seroprevalence rates of toxoplasmosis vary widely from 7 to 90% with regions of the Czech Republic [15]. We have currently no explanation for a significantly higher proportion of RhD-negative subjects in our sample of the ethnically highly homogenous Czech population. All study subjects were screened for health status prior to their enrolment in the study. In the informed consent form, the draftees were explained the general aim of the project (a study of influence of latent toxoplasmosis on human psychology and psychomotor performance) and the need for obtaining their consent to using results of their psychological and clinical examinations. About 80% of the conscripts consented to the use of their test results for the research project purposes and provided 5 ml of blood for serological testing. To keep the study blind, they were not informed about the *Toxoplasma *assay results and that the incidence rate of traffic accidents during their military service would be monitored. Subsequently, the data on *Toxoplasma *infection and RhD phenotype were matched with those on traffic accidents from military police records and then anonymized. The recruitment of study subjects and data handling were performed in compliance with Czech legislation in force and were approved by the Institutional Review Board of the Faculty of Science, Charles University.

### Serological tests

Serological tests for toxoplasmosis were carried out in the National Reference Laboratory for Toxoplasmosis of the National Institute of Public Health, Prague. Specific IgG and IgM antibody titers for toxoplasmosis were determined by ELISA (IgG: SEVAC, Prague, IgM: TestLine, Brno), optimized for the detection of acute toxoplasmosis [16], and complement fixation test (CFT) (SEVAC, Prague). The decrease in CFT titers is more regular and therefore better reflects the length of *T. gondii *infection [17]. CFT titers of antibodies to *Toxoplasma *in sera were measured at dilutions between 1:8 and 1:1024. For the purposes of this study, the subjects with negative results in the IgM ELISA (positivity index < 0.9) and an absorbance of > 0.250, i.e. approximately 10 IU/ml, in the IgG ELISA were considered latent toxoplasmosis positive while those with an absorbance of < 0.250 in the IgG ELISA were considered latent toxoplasmosis negative. Less than ten subjects with different diagnosis obtained with CFT and IgG ELISA tests or with suspected acute toxoplasmosis were excluded from the present study. The probability of false positive results in two independent serological tests is rather low, probably less than 3% [17]; however, theoretically, the population of seronegative subjects could be contaminated with an unknown (but probably very low) number of immunocompromised subjects [18] and subjects that acquired the infection during their military service. The RhD blood group type (presence of the RhD antigen on the erythrocyte membrane) was determined in all serum samples using the human monoclonal anti-D reagents (Seraclone^®^, ImmucorGamma Inc.) in the Central Military Hospital service laboratory.

### Statistical analysis

The Statistica 6.1 and SPSS 16.0 programs were used for statistical testing (frequency tables, logistic regression and Generalized linear model) and to check statistical tests assumptions. All variables including the covariates entered the respective analyses are specified in the Results section.

## Results

The rate of traffic accidents in 3890 male military conscripts was 2.85% (n = 111), see Table 1. In particular subgroups, the rates were as follows: 2.80% (*n *= 69) in 2460 RhD-positive *Toxoplasma*-free subjects, 2.40% (*n *= 17) in 709 RhD-positive *Toxoplasma*-infected subjects, 2.59% (*n *= 14) in 540 RhD-negative *Toxoplasma*-free subjects and 6.08% (n = 11) in 181 RhD-negative *Toxoplasma*-infected subjects. Analyses of frequency tables showed no significant effect of *Toxoplasma *(*Chi*^2 ^= 0.356, *P *= 0.551) or RhD phenotype (*Chi*^2 ^= 1.20, *P *= 0.273) on probability of traffic accidents. Logistic regression with dependent variable accident (binary) and independent factors toxo, RhD and toxo-RhD interaction showed significant effects of toxo (OR 2.43, *CI*_95_: 1.11–5.35, *t *= 2.157, *P *= 0.027) and toxo-RhD interaction (OR 0.35, *CI*_95_: 0.136–0.902, *t *= -2.17, *P *= 0.028) suggesting that *Toxoplasma *infection increased the chance of traffic accidents while RhD positivity reduced the risk of traffic accidents in *Toxoplasma*-infected subjects. To eliminate the effects of possible confounding variables driver's age and year of the beginning of military service, we included these variables into the model as independent factors. Again, the logistic regression showed significant effects of toxo (*OR *2.56, *CI*_95_: 1.14–5.76, *t *= 2.287, *P *= 0.022) and toxo-RhD interaction (*OR *0.35, *CI*_95_: 0.133–0.926, *t *= -2.12, *P *= 0.034). The effect of age was not significant (*OR *2.04, *CI*_95_: 0.47–8.92, *t *= 0.946, *P *= 0.344) and the effect of year of the beginning of military service was highly significant (*OR *2.85, *CI*_95_: 1.46–5.60, *t *= 3.052, *P *= 0.002). Separate logistic regression for RhD-negative subjects showed a 2.53 times higher risk of traffic accidents in *Toxoplasma*-infected than *Toxoplasma*-free subjects (*CI*_95_: 1.12–5.7, *t *= 2.23, *P *= 0.026). The same analysis for RhD-positive subjects showed no significant effect of *Toxoplasma *infection. Similarly, analysis for *Toxoplasma*-free subjects showed no significant effect of RhD phenotype, while this analysis for *Toxoplasma*-infected subjects revealed a 0.38 times lower risk of traffic accidents in RhD-positive than in RhD-negative subjects (*CI*_95_: 0.177–0.84, *t *= -2.39, *P *= 0.017).

**Table 1:** Number of subjects and age in particular categories.

Toxoplasmosis	Rh	Accident No		Accident Yes		Row	
No	negative	526 (13.52%)	20.23	14 (0.36%)	20.64	540 (13.88%)	20.24

No	positive	2391 (61.47%)	20.17	69 (1.77%)	20.29	2460 (63.24%)	20.17

Total		2917 (74.99%)	20.18	83 (2.13%)	20.35	3000 (77.12%)	20.18

Yes	negative	170 (4.37%)	19.98	11 (0.28%)	19.91	181 (4.65%)	19.97

Yes	positive	692 (17.79%)	20.02	17 (0.44%)	20.65	709 (18.23%)	20.04

Total		862 (22.16%)	20.01	28 (0.72%)	20.36	890 (22.88%)	20.02

Column Total		3779 (97.15%)	20.14	111 (2.85%)	20.35	3890 (100%)	20.15

**Figure 1:**
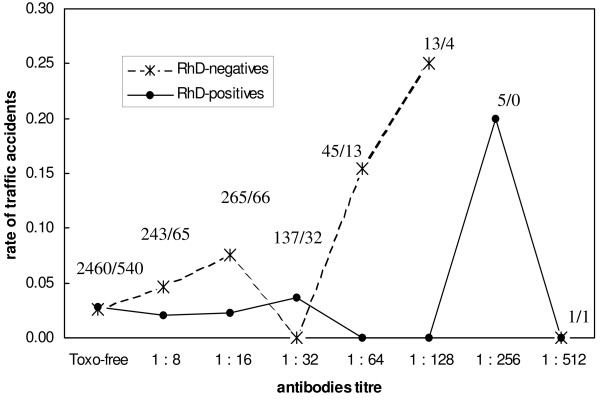
**Rates of traffic accidents in RhD-negative (crosses) and RhD-positive (circles) military drivers within 12–18 months of military service**. The y-axis shows rate (probability) of traffic accidents for subjects with particular anti-*Toxoplasma *antibody titers. The figures indicate numbers of RhD-positive/RhD-negative subjects with particular anti-*Toxoplasma *antibody titers (x-axis).

To search for possible effect of anti-*Toxoplasma *antibody titer (indication of the length of the infection) on risk of traffic accidents, we analysed *Toxoplasma*-infected subjects using Generalized linear model with independent variable accident and independent binary variable RhD and ordinal variable titer of antibodies (probability distribution: binary, link function: logit). The results showed a significant effect of interaction RhD-titre of antibodies (*Chi*^2 ^= 7.85, *P *= 0.02). The chance of traffic accidents increased 1.42 times (*CI*_95_: 0.01–0.68) for each titration step in RhD-negative subjects (*B *= 0.35, *CI*_95_: 0.01–0.68, *Chi*^2 ^= 4.18, *P *= 0.041). However, no effect of antibody titer was observed in more numerous RhD-positive subjects (*B *= 0.07, *CI*_95_: -0.27–0.4.25, *Chi*^2 ^= 0.18, *P *= 0.674), Fig. 1.

## Discussion

Our results show that *Toxoplasma *infection increased the risk of traffic accidents in military drivers. The data also indicate that RhD positivity protected infected subjects against the increase in the rate of traffic accidents. In RhD-negative subjects, the probability of traffic accidents increased with titer of anti-*Toxoplasma *antibodies suggesting that the risk of traffic accidents decreased with duration of *Toxoplasma *infection. The probability of traffic accidents increased across the years of testing. This phenomenon was, probably, caused by the observed downward trend in overall quality, e.g. in intelligence, of conscripts (data not shown), related to the forthcoming abolition of compulsory military service in the Czech Republic in 2004.

Our results are in good agreement with the results of laboratory testing of reaction times in *Toxoplasma*-infected and *Toxoplasma*-free, RhD-positive and RhD-negative subjects [6,9,10]. Latent toxoplasmosis had a strong negative effect on RhD-negative subjects and no effect on RhD-positive subjects. The strength of the negative effect of toxoplasmosis on reaction times increased with duration of infection (with decreasing anti-*Toxoplasma *antibody titer) [6]. In contrast, in the current study the risk of traffic accidents decreased with duration of infection. It should be noted, however, that the decrease of the risk of traffic accidents with duration of infection was already observed in a previous case-control study [7]. The most plausible explanation for such conflicting results is that, on the one hand, reaction times increase with duration of infection and, on the other hand, the infected drivers are able to gradually adapt to their impaired reaction times. Such effect has already been described in aging drivers [19].

It was previously speculated that personality differences between *Toxoplasma*-infected and *Toxoplasma*-free subjects could result in lower willingness of *Toxoplasma*-infected subjects to participate in serological surveys and therefore also in their underrepresentation in a control population [7]. The design of the current study, i.e. a prospective cohort study, made it possible to confirm that the increase of the risk of being involved in a traffic accident rather than a decreased chance of being enrolled in epidemiological surveys is responsible for the higher prevalence of *Toxoplasma*-infected subjects among victims of traffic accidents compared to the general population of the same area.

At face value, our results in some respect contradict those of the previous traffic accident studies. In the present study, the effect of latent toxoplasmosis on traffic accidents was not apparent in an unsorted population of RhD-positive and RhD-negative subjects. When infected with *Toxoplasma*, the RhD-negative subjects had a significantly higher and RhD-positive subjects a nonsignificantly lower incidence rate of traffic accidents than the corresponding *Toxoplasma*-free subjects. On the other hand, the present results are in agreement with the unpublished results of our retrospective case-control study performed in Prague between 2002–2006 using the same method as in the 1997–2000 study [7]. In that study, similarly as in the present prospective study, the effect of latent toxoplasmosis on the incidence rate of traffic accidents in the RhD unsorted population was not significant. In January 2000, a dramatic change in Czech traffic rules came into action. For example, pedestrians did not have priority at zebra crossings before the year 2000. We suppose that changes in behaviour of Czech drivers and pedestrians could be responsible for differences between results from 1997–2000 and 2001–2006.

It must also be noted that the effect of latent toxoplasmosis on reaction times varies across testing minutes [6,9,10]. The effect of toxoplasmosis on reaction time is always stronger in RhD-negative subjects than in RhD-positive subjects. However, RhD-negative *Toxoplasma*-infected men expressed nonsignificantly shorter reaction times (p = 0.37) than RhD-negative *Toxoplasma*-free subjects in the first minute of the three minute-test [10]. The authors speculated that two effects of infection might interplay in *Toxoplasma*-infected men. Latent toxoplasmosis is known 1) to impair reaction times in infected men [6] and animals [20] and also 2) to increase the concentration of testosterone in infected men [21-23]. Increased concentration of testosterone positively influences the level of personality trait competitiveness in men [24], which, in turn, could enhance performance under certain circumstances. Therefore, the negative effects of latent toxoplasmosis on psychomotor performance in men could influence the rate of traffic accidents under certain conditions only, which could explain some differences in results between the published case-control studies and present cohort study.

Our study has several limitations. Despite the fact that nearly 80% of draftees agreed to participate in our study, this population cannot be considered fully representative of the Czech general population. For example, our anecdotal observation of increased frequency of traffic accidents in 20% of drivers who did not agree to participate, suggested that this subpopulation differed in both psychological profile and probability of traffic accident from subpopulation under the study.

The subjects were screened for latent toxoplasmosis in the beginning of the military service only. Some drivers probably acquired toxoplasmosis during their 0.5–1.5 year military service. It must be stressed, however, that the presence of *Toxoplasma*-infected drivers in our set of *Toxoplasma*-free subjects (as well as possible presence of *Toxoplasma*-free drivers in our set of *Toxoplasma*-infected subjects) can only increase risk of false negative but not false positive results of our statistical tests.

According to military legislation all traffic accidents including material damage and/or injuries shall be reported to the military police. Of course, some less serious accidents might remain unreported; however, according to the experience of military traffic experts completeness of military records is much higher in comparison with the civic sphere (personal communication).

A major limitation of the present study is the absence of RHD genotype data. Previous case-control study on a large sample of blood donors has shown that RhD-positive heterozygotes are resistant to pathological effects of toxoplasmosis while RhD-positive homozygotes are only temporarily resistant: their psychomotor performance decreases with length of infection. The psychomotor performance of RhD-negative homozygotes decreases immediately after infection. Our population of RhD-positive drivers includes both RhD-positive heterozygotes and RhD-positive homozygotes (some of them with relatively low concentration of anti-*Toxoplasma *antibodies and therefore relatively old infection). Possible contamination of the accident protected RhD population by an unknown number of non-protected RhD-homozygotes might decrease the power of our tests and undervalue the strength of the observed effects.

The absence of data on *RHD *genotype of subjects makes any speculation on the mechanism of RhD positivity protection very difficult. However, the results of previous studies [6,9,10] and current knowledge about localization and probable function of RH proteins [11,12] suggest that RhD and RhCE proteins that act as ion pumps, coded for at the RH locus and localized on the erythrocyte membrane, are involved in the regulation of ion balance in some critical compartment of nerve or muscle tissue. Such regulation could be important especially in the subjects handicapped by the presence of *Toxoplasma *cysts in nerve and muscle tissues.

## Conclusion

The influence of recent (subacute) *Toxoplasma *infection on the rate of traffic accidents is relatively strong. Three of seventeen RhD-negative military drivers (16.7%) with anti-*Toxoplasma *antibody titers of 1:64 or higher, i.e. the subjects likely to be infected with *Toxoplasma *for less than two years [17], had a traffic accident within the next 12–18 months while the risk rate in other groups of military drivers was about 2.6%. We suggest that RhD-negative drivers diagnosed with acute and subacute toxoplasmosis should be informed about the transiently increased risk of traffic accidents.

The observed effects could provide not only a clue to the long-standing evolutionary enigma of the origin of RhD polymorphism in humans (the effect of balancing selection), but might also be the missing piece in the puzzle of the physiological function of the RhD molecule.

## Competing interests

The authors declare that they have no competing interests.

## Authors' contributions

JF conceived of the study, participated in its design and coordination, and performed all statistical analyses. JK participated in the design of the study and organized collection of data at Central Military Hospital. MN, MB and JH collected the data and participated in writing the manuscript. All authors read and approved the final manuscript.

## Pre-publication history

The pre-publication history for this paper can be accessed here:

http://www.biomedcentral.com/1471-2334/9/72/prepub
